# Clinical and microbiological determinants of treatment failure in complicated urinary tract infection: a four-hospital cohort of enterobacterales infections in Misurata, Libya

**DOI:** 10.3389/fmed.2026.2002811

**Published:** 2026-10-19

**Authors:** Mohanned Mohamed Alwashaish, Adel Omar Ashour, Marwa Elsadeq Elwash, Fathia Muftah Elmeheishi

**Affiliations:** 1 Department of Biomedical Sciences, Faculty of Pharmacy, Misurata University, Misurata, Libya; 2 Department of Microbiology, Faculty of Science, Misurata University, Misurata, Libya; 3 Department of Genetics and Biotechnology, Faculty of Science, Misurata University, Misurata, Libya

**Keywords:** antimicrobial resistance, beta-lactamase co-production, biofilm, complicated urinary tract infection, enterobacterales, Libya, treatment failure

## Abstract

**Background:**

Complicated urinary tract infection (cUTI) is influenced by host vulnerability, urinary tract abnormalities, antimicrobial resistance, and bacterial persistence. We evaluated whether phenotypic beta-lactamase co-production and biofilm formation were independently associated with treatment failure among adults with Enterobacterales cUTI in Misurata, Libya.

**Methods:**

This prospective multicenter cohort included 963 cUTI episodes and 1,017 Enterobacterales isolates collected continuously at four hospitals from 1 March 2024 through 31 March 2026. Identification combined conventional biochemical testing, API 20E, and MALDI Biotyper confirmation. Antimicrobial susceptibility testing used CLSI disk diffusion; selected isolates underwent agar-dilution minimum inhibitory concentration testing, phenotypic beta-lactamase testing, and triplicate microtiter-plate biofilm assessment. The prespecified composite outcome was reconciled against its recorded component fields before modeling; documented same-organism persistence at 3–7 days constituted treatment failure under the prespecified rule. Multivariable logistic regression included prespecified covariates and hospital fixed effects and was used for explanatory association estimation rather than clinical prediction.

**Results:**

Rule-based outcome reconciliation identified 188/963 treatment failures (19.5%), including 36 episodes with same-organism persistence that had been discordantly coded as successes. Obstruction (adjusted odds ratio [aOR] 1.55, 95% CI 1.06-2.26), diabetes (aOR 1.48, 95% CI 1.06-2.09), and sepsis (aOR 1.59, 95% CI 1.10-2.29) were associated with failure after adjustment. Phenotypic beta-lactamase co-production was not independently associated with failure (aOR 0.62, 95% CI 0.35-1.09). Biofilm positivity showed an inverse association in the primary model (aOR 0.70, 95% CI 0.49-0.99), but this finding was not robust across sensitivity analyses. In a *post hoc* sensitivity analysis excluding microbiological persistence from the composite outcome (147 events), neither co-production (aOR 0.53, 95% CI 0.27-1.03) nor biofilm positivity (aOR 0.76, 95% CI 0.51-1.13) was statistically significant.

**Conclusion:**

Host severity and remediable urinary-tract factors showed more consistent associations with treatment failure than the microbiological phenotypes evaluated. The inverse biofilm pattern is observational, sensitive to outcome definition, and should not be interpreted as protective or causal; the internally corrected model had limited discrimination and was not developed as a prediction tool.

## 1 Introduction

Complicated urinary tract infections comprise a heterogeneous group of infections occurring in the presence of structural, functional, or host factors that increase the probability of adverse outcomes. Contemporary reviews and guidance emphasize that obstruction, urinary devices, renal dysfunction, diabetes, systemic illness, and antimicrobial resistance can alter both treatment selection and prognosis (1–5).

The growing burden of resistant Enterobacterales is especially important in settings where empiric therapy is constrained by high rates of extended-spectrum beta-lactamase (ESBL), AmpC beta-lactamase, carbapenemase, fluoroquinolone resistance, and multidrug resistance. Recent therapeutic guidance therefore links organism phenotype, infection severity, source control, and achievable urinary drug concentrations rather than treating resistance as an isolated laboratory result (6–9). African surveillance studies also show substantial geographic variation, reinforcing the need for locally generated clinical-microbiological data (10–15).

Biofilm formation provides a plausible mechanism for persistence, particularly in catheterized or structurally abnormal urinary tracts, and has been linked with uropathogenicity and antimicrobial tolerance in laboratory studies (16–20). However, isolate-level biofilm capacity does not necessarily translate into episode-level treatment failure after accounting for host severity, urinary obstruction, therapy, and source control. Similarly, co-production of beta-lactamases may broaden resistance phenotypes but its independent clinical contribution in cUTI remains insufficiently characterized.

We therefore evaluated the clinical, microbiological, and treatment-related determinants of treatment failure in a four-hospital cohort of Enterobacterales cUTI in Misurata. The primary hypothesis was that beta-lactamase co-production and biofilm formation would be associated with treatment failure after prespecified adjustment. Secondary objectives were to describe the isolate distribution, resistance phenotypes, antimicrobial susceptibility profile, and robustness of the primary associations across clinically relevant sensitivity analyses.

## 2 Materials and methods

### 2.1 Study design and setting

We conducted a prospective multicenter cohort study at Misurata Medical Center (public, 1,300 beds), Alshifa Hospital (private, 86 beds), Albanon Hospital (private, 131 beds), and Alsafwa Hospital (private, 160 beds), Misurata, Libya. Eligible episodes were enrolled and processed in real time from 1 March 2024 through 31 March 2026. The episode was the primary unit of clinical analysis and the bacterial isolate was the unit of laboratory analysis. The report follows the STROBE recommendations for cohort studies (21).

### 2.2 Eligibility and episode definition

Eligible episodes involved adults aged 18 years or older with signs or symptoms compatible with UTI, a quantitatively significant urine culture yielding at least one Enterobacterales isolate, and at least one complicating host or urinary-tract factor. Complicating criteria included male sex, pregnancy, urinary catheterization, obstruction, urolithiasis, structural or functional abnormality, neurogenic bladder, recent urological procedure, immunocompromise, clinically relevant comorbidity, or healthcare association. These criteria reflect contemporary cUTI concepts that distinguish localized uncomplicated infection from infection complicated by systemic illness or factors that jeopardize treatment success (1, 2, 5). Only outcome-assessable episodes were included. Each episode received a unique code, and every clinically significant Enterobacterales isolate from polymicrobial cultures received a linked isolate code.

### 2.3 Clinical variables and outcome

Demographic characteristics, hospital and service, healthcare association, urinary-tract factors, prior healthcare and antimicrobial exposures, comorbidities, Charlson score, clinical severity, inflammatory and renal indices, culture characteristics, antimicrobial treatment, time to active therapy, source-control requirements, and 30-day outcomes were recorded prospectively on standardized forms. Treatment failure was a prespecified composite intended to capture clinically important non-resolution or worsening across complementary domains rather than to imply that all components represented the same biological process. It comprised: (i) persistent or worsening cUTI signs or symptoms at 72 h that required continued or escalated management; (ii) microbiological persistence of the same clinically significant organism in a repeat urine culture obtained 3-7 days after the index culture; (iii) documented antimicrobial escalation because of clinical non-response; or (iv) ICU transfer during the index episode, recurrence or UTI-related readmission within 30 days, or death within 30 days. Components were not mutually exclusive, and component-level counts are reported separately in Supplementary Table S4. Clinical deterioration was not captured as a separate structured binary field; severe deterioration was represented by the recorded ICU-transfer and other structured clinical-course components. Mortality attribution to cUTI was not reliably classifiable in the source dataset, so 30-day all-cause mortality is reported descriptively rather than as a separately attributed UTI-specific endpoint. The component variables were recorded before final analysis and were not entered as predictors of the composite outcome. Before modeling, the locked outcome file was reconciled against the prespecified component rule as a data-quality step rather than as a new clinical adjudication. Two authors (MA and AA) independently cross-checked the locked outcome coding against the prespecified recorded component fields. This was not an exposure-blinded clinical adjudication, and no adjudication committee external to the author team was used; rather, the task was a deterministic verification of whether the recorded component fields satisfied the prespecified rule. Thirty-six episodes with documented same-organism persistence at 3-7 days had been discordantly coded as successes and were corrected to failure; no new outcome component was introduced. All 36 were identified because same-organism persistence was the qualifying component for the correction; none had recorded 72-hour non-response, escalation due to failure, ICU transfer, recurrence, readmission, or death. Three had persistent symptoms recorded, but their 72-hour clinical-response field was ‘Improved' and no escalation was recorded, so those symptoms did not independently satisfy the prespecified clinical non-response component. The primary model was also repeated using the original 152-event coding to assess sensitivity to the data-quality correction. In addition, a *post hoc* sensitivity outcome was constructed with the microbiological-persistence component omitted entirely; episodes were retained as failures only when at least one non-microbiological qualifying component remained. This produced 147 failures. The same prespecified covariate set was refit unchanged. The 72-hour assessment is consistent with recent work using early non-response composites in hospitalized UTI (22).

### 2.4 Microbiological identification and susceptibility testing

Enterobacterales were provisionally identified from colony morphology on primary and differential media and a conventional biochemical panel comprising lactose fermentation, oxidase, indole, methyl red, Voges-Proskauer, citrate, urease, triple sugar iron slant/butt reactions, gas and hydrogen sulfide production, motility, lysine and ornithine decarboxylation, and phenylalanine deamination as appropriate. The biochemical profile was then evaluated with API 20E identification strips (bioMérieux, Marcy-l'Étoile, France) according to the manufacturer instructions. Species identity was confirmed using the MALDI Biotyper system (Bruker Daltonics, Bremen, Germany), with spectra interpreted using the manufacturer acceptance criteria and only concordant high-confidence identifications released for analysis. The specific MALDI instrument model and the software/database version identifiers were not retained in the study dataset and were not reconstructed retrospectively; discordant or low-confidence identifications were resolved before release by repeat phenotypic/API assessment and MALDI confirmation rather than by assigning an uncertain species call.

Disk diffusion was performed prospectively on Mueller-Hinton agar in accordance with CLSI M02 (23). A fresh pure culture was suspended to 0.5 McFarland turbidity, inoculated over the complete agar surface in three directions, and disks were applied promptly. Plates were incubated aerobically at 35 ± 2 °C for 16–18 h. The panel comprised ampicillin 10 micrograms, amoxicillin-clavulanate 20/10 micrograms, piperacillin-tazobactam 100/10 micrograms, cefotaxime 30 micrograms, ceftazidime 30 micrograms, cefepime 30 micrograms, ertapenem 10 micrograms, imipenem 10 micrograms, meropenem 10 micrograms, gentamicin 10 micrograms, amikacin 30 micrograms, ciprofloxacin 5 micrograms, trimethoprim-sulfamethoxazole 1.25/23.75 micrograms, nitrofurantoin 300 micrograms, and fosfomycin 200 micrograms plus 50 micrograms glucose-6-phosphate. Inhibition-zone diameters were measured to the nearest millimeter and retained as raw measurements. Because enrollment began before publication of CLSI M100 Ed36, susceptibility testing was performed in real time using CLSI disk-diffusion procedures and contemporaneous quality control; for the final harmonized analysis, all stored zone diameters were reinterpreted using species-specific CLSI M100 Ed36 (2026) breakpoints (24). Thus, Ed36 was applied retrospectively to the recorded measurements at final analysis rather than being claimed as the interpretive edition used throughout 2024–2026. *Escherichia coli* ATCC 25922 was included for quality control; runs outside the accepted QC range were not released. Nitrofurantoin and fosfomycin were interpreted only for supported species and indications.

Multidrug resistance (MDR) was defined as acquired non-susceptibility to at least one agent in at least three antimicrobial categories, following the international interim definitions (25). Intermediate and susceptible-dose-dependent results were retained as separate categories in the antibiogram and treated as non-susceptible only where required by the MDR category definition.

### 2.5 Phenotypic beta lactamase testing

ESBL screening used cefotaxime 30-microgram and ceftazidime 30-microgram disks. Phenotypic confirmation used a double-disk synergy arrangement with an amoxicillin-clavulanate 20/10-microgram disk placed centrally and the cefotaxime and ceftazidime disks positioned 20 mm from the center of the amoxicillin-clavulanate disk. Enhancement or keyhole expansion of either cephalosporin inhibition zone toward amoxicillin-clavulanate was interpreted as synergy according to the prespecified protocol, which was based on the double-disk synergy principle (26). Because this was a local central-disk implementation, it is described explicitly rather than mislabeled as a molecular or combination-disk test.

AmpC screening used cefoxitin 30 micrograms, followed by a Tris-EDTA AmpC disk test; indentation or expansion of the cefoxitin inhibition zone adjacent to the enzyme-extraction disk was interpreted as positive (27). Carbapenemase phenotyping was performed for all Enterobacterales isolates using the modified carbapenem inactivation method (mCIM), with interpretation according to CLSI criteria (24, 28). Isolates with a positive mCIM result were classified as phenotypic carbapenemase producers. Among mCIM-positive isolates, metallo-beta-lactamase activity was further assessed using imipenem and imipenem-EDTA disks in parallel, and the EDTA-associated increase in inhibition zone was recorded (29). Two independent readers recorded phenotypic results and discrepancies were resolved before data entry. For the prespecified exposure, beta-lactamase co-production was defined operationally as two or more confirmed phenotypic beta-lactamase categories in the same isolate. This threshold was chosen to distinguish isolates expressing overlapping phenotypic resistance mechanisms from isolates with a single detected category; it is an epidemiological phenotype definition and does not imply co-carriage of specific resistance genes or enzymes.

### 2.6 Minimum inhibitory concentration testing

Agar-dilution MIC testing followed the isolate-selection rule recorded before outcome modeling. Meropenem MIC was measured for all 107 carbapenemase-positive isolates and for 117 additional carbapenemase-negative isolates selected from the remaining collection according to the laboratory MIC sampling plan, representing approximately 13% of that pool and without selection on treatment outcome; this produced 224 meropenem MIC measurements. Cefepime MIC was measured only for the 107 carbapenemase-positive isolates. The exact selection counts and rule are reported in Supplementary Table S1.

The procedure followed CLSI M07 (30). Twofold antimicrobial concentrations were incorporated into Mueller-Hinton agar. Standardized suspensions delivered approximately 10^4 CFU per inoculated spot. Growth and sterility controls were included, and E. coli ATCC 25922 served as the quality-control strain. Plates were incubated aerobically at 35 ± 2 °C for 16–20 h. The MIC was defined as the lowest concentration showing complete inhibition of visible growth. Raw MIC values were retained and, as with disk diffusion, final categorical interpretations were harmonized using CLSI M100 Ed36 (24). MIC50 and MIC90 were defined as the lowest concentrations inhibiting at least 50% and 90% of tested isolates, respectively. Because sampling was intentionally enriched for carbapenemase-positive isolates, MIC distributions were not generalized to the full isolate collection.

### 2.7 Biofilm assay

Biofilm formation was quantified using the microtiter-plate crystal-violet procedure described by Stepanovic et al. (31), with three technical replicate wells per isolate. A fresh 0.5 McFarland bacterial suspension was inoculated as 20 microliters into 180 microliters of tryptic soy broth supplemented with 1% glucose (final volume 200 microliters per well); negative-control wells contained 200 microliters of uninoculated supplemented broth. Plates were incubated aerobically under static conditions for 24 h at 35–37 degrees C. After incubation, well contents were discarded and each well was washed three times with 300 microliters of sterile phosphate-buffered saline (approximately pH 7). Adherent biomass was fixed with 150 microliters of methanol for 20 min, the plates were emptied and air-dried, and wells were stained at room temperature with 150 microliters of 2% Hucker crystal violet for 15 min. Unbound stain was removed by rinsing with water until washings were clear, followed by air drying. Bound dye was resolubilized with 150 microliters of 95% ethanol and optical density was read at 570 nm. Each plate run included triplicate negative controls and an E. coli positive control. The optical-density cutoff (ODc) was calculated separately for each valid plate as the mean negative-control optical density plus three standard deviations. Following the Stepanovic classification (31), isolates were categorized as non-producers (OD <= ODc), weak producers (ODc < OD <= 2xODc), moderate producers (2xODc < OD <= 4xODc), or strong producers (OD > 4xODc). The arithmetic mean of the three technical wells was used for classification; technical wells were not treated as independent biological replicates. Laboratory records show one validated plate run per isolate set, with invalid-control runs repeated; isolates were not routinely retested on separate days as independent biological replicates. The positive control served as a qualitative process control, whereas ODc and category assignment were determined from negative-control measurements; no formal inter-run reproducibility statistic or quantitative positive-control acceptance range was prespecified. Episode-level biofilm positivity was present when at least one linked Enterobacterales isolate was positive.

### 2.8 Exposure definitions for polymicrobial episodes

For the primary episode-level analysis, co-production, ESBL, AmpC, carbapenemase, and biofilm exposures were considered present if at least one clinically significant linked Enterobacterales isolate expressed the phenotype. This prespecified any-isolate rule was used because the clinical unit of analysis was the infection episode and, in polymicrobial cultures, a single clinically significant resistant or biofilm-positive Enterobacterales isolate could plausibly influence antimicrobial selection or persistence. The rule does not identify which organism was the dominant causal contributor and can therefore introduce episode-level exposure misclassification when linked isolates differ in phenotype. Polymicrobial status, the number of Enterobacterales isolates, and non-Enterobacterales co-isolates remained episode-level variables. A monomicrobial-only sensitivity analysis was used to assess robustness to the any-isolate definition.

### 2.9 Study size

The minimum study size was evaluated primarily for precision and was supplemented by prespecified model-design considerations. A 20% treatment-failure proportion was used as a planning value for the precision calculation; this value was not estimated from the present cohort and is consistent with earlier cUTI cohorts reporting treatment-failure frequencies in approximately the 20%–30% range (32). With 95% confidence and absolute precision of 3%, *n* = Z^2 p(1-p)/d^2 yielded 683 episodes. Allowing for up to 20% incomplete follow-up by dividing by 0.80 produced a target of 854 episodes (33). This calculation is presented as a study-size planning calculation rather than as a *post hoc* derivation from the observed 19.5% event rate. The final cohort of 963 exceeded the target. The primary model contained 15 non-intercept parameters and 188 reconciled failures (12.5 events per parameter); this ratio is reported descriptively and is not treated as definitive evidence of model adequacy. Model stability was instead considered together with prespecification, collinearity assessment, sensitivity analyses, and bootstrap internal validation.

### 2.10 Statistical analysis

Primary analyses were specified for IBM SPSS Statistics version 26; reproducibility checks, bootstrap validation, influence diagnostics, heatmaps, and Firth penalized sensitivity analysis were performed with independently scripted numerical routines. Categorical variables were summarized as *n* (%) and compared using Pearson chi-square or Fisher exact tests. Continuous variables were summarized as median and interquartile range and compared using the Mann–Whitney U test. Species-specific enzyme frequencies used the species count as denominator. Species-specific susceptibility percentages used the number of isolates with an interpretable result for that species-agent combination; Not_applicable results were reported separately and excluded from the denominator.

The primary binary logistic regression estimated aORs, 95% CIs, and two-sided *p* values for the reconciled treatment-failure outcome. Co-production and biofilm were forced into the model. Prespecified adjustment included age, sex, hospital fixed effects, urinary catheterization, obstruction, prior antimicrobial exposure, diabetes, chronic kidney disease, Charlson score, sepsis, and polymicrobial infection. Covariates were selected by clinical relevance rather than automated screening. Tolerance and VIF assessed collinearity. Discrimination was evaluated using the area under the receiver-operating-characteristic curve (AUC) with a nonparametric bootstrap 95% CI. Calibration was summarized by the apparent in-sample calibration intercept and slope, decile calibration plot, Hosmer-Lemeshow statistic, and Brier score; internal validation used 10,000 bootstrap resamples to estimate optimism in both AUC and calibration slope. Approximate Cook distance identified influential observations, with 4/n used as a screening threshold rather than an automatic deletion rule. The model was specified for explanatory association estimation and internal stability assessment, not for development of a clinical prediction rule.

Secondary models evaluated the co-production-by-biofilm interaction, ordinal biofilm intensity, and episode-level MDR. The treatment-extended model represented source control as three mutually exclusive states: not required, required and adequate, and required but inadequate; it also included empiric *in-vitro* inactivity and time to active therapy. Prespecified sensitivity analyses were restricted to monomicrobial episodes, *Escherichia coli*/*Klebsiella pneumoniae* episodes, and episodes without carbapenemase-positive isolates. A Firth penalized logistic model assessed small-sample bias for co-production, and an additional outcome-coding sensitivity model used the original 152-event coding before correction of the 36 discordant records. Separately, a *post hoc* component-deletion sensitivity analysis omitted microbiological persistence from the composite outcome and refit the unchanged prespecified primary covariate set to the resulting 147-event outcome. Repeat urine cultures were clinically indicated rather than protocol-mandated; verification-bias analyses therefore compared cultured and non-cultured episodes descriptively. Unknown, Not_done, Not_cultured, Not_applicable, and true missing values were retained as distinct states. Statistical significance was set at *p* < 0.05, but secondary and sensitivity analyses were treated as exploratory and interpreted primarily through effect estimates, confidence intervals, and cross-analysis consistency rather than isolated *p* values.

### 2.11 Ethics statement

The study protocol was approved on 13 February 2024 by the Research Ethics Committee of the Faculty of Pharmacy, Misurata University (approval no. 81/2024), before enrollment began on 1 March 2024. The committee explicitly approved verbal informed consent, waived the requirement for a written participant signature, and approved use of coded clinical and laboratory data without names or direct personal identifiers. Verbal informed consent was obtained before enrollment, and clinical management was not altered by study participation.

## 3 Results

### 3.1 Cohort and outcome

All 963 outcome-assessable episodes were included. Median age was 56 years (IQR 37–76), 615 (63.9%) patients were female, and 318 (33.0%) had a urinary catheter at culture. Rule-based reconciliation identified 36 records with documented same-organism persistence at 3–7 days that had been coded as successes, increasing failures from 152 to 188 (19.5%); 775 episodes were classified as success. The 36 corrections were triggered by the same-organism persistence component: none had recorded 72-hour non-response, escalation due to failure, ICU transfer, recurrence, readmission, or death. No duplicate episode or isolate identifiers, broken episode-isolate links, or isolate-count mismatches were detected. The cohort flow is shown in Figure 1, and baseline characteristics according to treatment outcome are summarized in Table 1. Obstruction, diabetes, and sepsis were more common among episodes with failure, whereas age, sex, catheterization, prior antibiotic exposure, chronic kidney disease, and polymicrobial infection did not differ materially in unadjusted comparisons.

**Figure 1:**
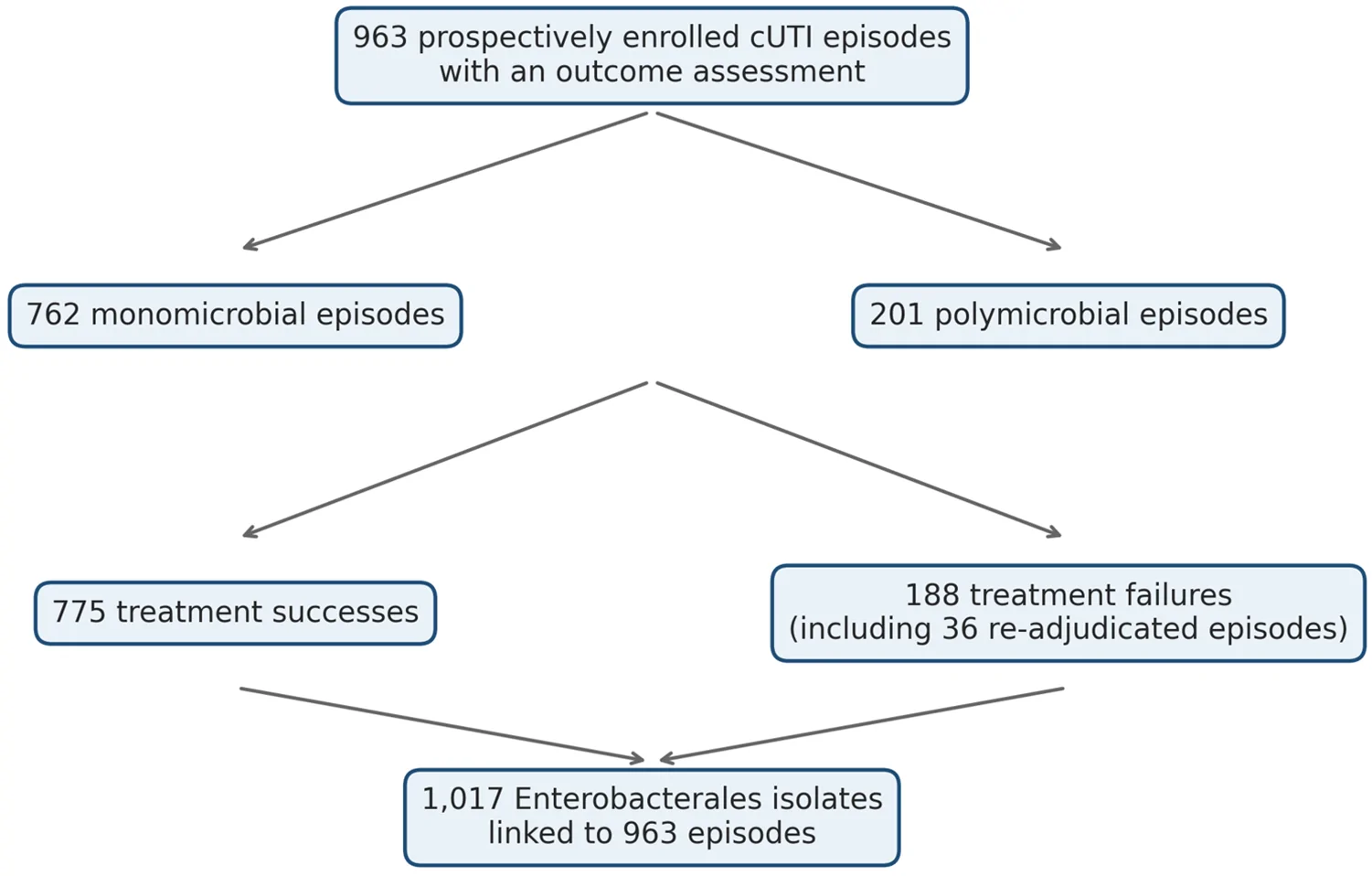
Flow of episodes and enterobacterales isolates through the clinical and laboratory analyses after rule-based outcome reconciliation.

**Table 1:** Baseline characteristics according to treatment outcome.

Characteristic	Overall *n* = 963	Success *n* = 775	Failure *n* = 188	p
Age years	56.0 (37.0–76.0)	56.0 (37.0–75.5)	55.0 (37.0–76.0)	0.964
Female sex	615 (63.9)	491 (63.4)	124 (66.0)	0.554
Healthcare associated	399 (41.4)	321 (41.4)	78 (41.5)	1.000
Hospital onset	305 (31.7)	248 (32.0)	57 (30.3)	0.727
Urinary catheter	318 (33.0)	256 (33.0)	62 (33.0)	1.000
Obstruction	196 (20.4)	147 (19.0)	49 (26.1)	0.034
Urolithiasis	181 (18.8)	143 (18.5)	38 (20.2)	0.603
Structural abnormality	183 (19.0)	151 (19.5)	32 (17.0)	0.470
Prior antibiotics within 90 days	411 (42.7)	331 (42.7)	80 (42.6)	1.000
Diabetes	293 (30.4)	223 (28.8)	70 (37.2)	0.027
Chronic kidney disease	165 (17.1)	126 (16.3)	39 (20.7)	0.160
Charlson score	4.0 (2.0–6.0)	4.0 (2.0–6.0)	4.0 (2.0–6.0)	0.701
Sepsis	217 (22.5)	162 (20.9)	55 (29.3)	0.019
Bacteremia with same organism	84 (8.7)	58 (7.5)	26 (13.8)	0.001

### 3.2 Microbiology and resistance phenotypes

The 963 episodes yielded 1,017 Enterobacterales isolates. Species-specific *β*-lactamase phenotypes are summarized in Table 2. *E. coli* accounted for 482 (47.4%), *K. pneumoniae* for 221 (21.7%), *Proteus mirabilis* for 112 (11.0%), *Enterobacter cloacae* complex for 57 (5.6%), and *Citrobacter freundii* complex for 38 (3.7%). ESBL production was confirmed in 372 isolates (36.6%), AmpC in 180 (17.7%), carbapenemase in 107 (10.5%), and two or more beta-lactamase phenotypes in 112 (11.0%). Biofilm formation was detected in 732 isolates (72.0%): 229 weak, 311 moderate, and 192 strong producers. MDR was present in 559 isolates (55.0%).

**Table 2:** Species-specific beta-lactamase phenotypes among 1,017 enterobacterales isolates.

Species	n	ESBL *n* (%)	AmpC *n* (%)	Carbapenemase *n* (%)	Co-production *n* (%)
*Escherichia coli*	482	211 (43.8)	64 (13.3)	53 (11.0)	57 (11.8)
*Klebsiella pneumoniae*	221	94 (42.5)	23 (10.4)	25 (11.3)	26 (11.8)
*Proteus mirabilis*	112	22 (19.6)	17 (15.2)	10 (8.9)	4 (3.6)
*Enterobacter cloacae complex*	57	14 (24.6)	30 (52.6)	8 (14.0)	12 (21.1)
*Citrobacter freundii complex*	38	4 (10.5)	19 (50.0)	6 (15.8)	6 (15.8)
*Morganella morganii*	29	11 (37.9)	3 (10.3)	1 (3.4)	1 (3.4)
*Serratia marcescens*	24	4 (16.7)	12 (50.0)	3 (12.5)	4 (16.7)
*Klebsiella aerogenes*	23	2 (8.7)	8 (34.8)	0 (0.0)	0 (0.0)
*Providencia stuartii*	19	5 (26.3)	1 (5.3)	1 (5.3)	1 (5.3)
*Citrobacter koseri*	12	5 (41.7)	3 (25.0)	0 (0.0)	1 (8.3)

The distribution of enzyme phenotypes differed markedly by species. ESBL production was concentrated in *E. coli* (211/482, 43.8%) and *K. pneumoniae* (94/221, 42.5%), whereas AmpC was most frequent in *E. cloacae* complex (30/57, 52.6%), *C. freundii* complex (19/38, 50.0%), and *Serratia marcescens* (12/24, 50.0%). Co-production reached 12/57 (21.1%) in *E. cloacae* complex and 6/38 (15.8%) in *C. freundii* complex, compared with 57/482 (11.8%) in *E. coli*, 26/221 (11.8%) in *K. pneumoniae*, and 4/112 (3.6%) in *P. mirabilis*. Complete combinations are reported in Supplementary Table S2.

Resistance was species dependent. In *E. coli*, 252/482 (52.3%) were resistant to cefotaxime, 249/482 (51.7%) to ceftazidime, 241/482 (50.0%) to ampicillin, and 231/482 (47.9%) to amoxicillin-clavulanate. Corresponding resistance in *K. pneumoniae* was 109/221 (49.3%) to cefotaxime, 105/221 (47.5%) to ceftazidime, and 103/221 (46.6%) to amoxicillin-clavulanate. *E. cloacae* complex showed 33/57 (57.9%) resistance to both ertapenem and ampicillin, while *C. freundii* complex showed 20/38 (52.6%) resistance to ceftazidime and 19/38 (50.0%) to cefepime. Figure 2 displays all ten recovered species across all 15 agents. Supplementary Table S3 provides tested denominators and complete S, I/SDD, R, and Not applicable counts and percentages.

**Figure 2:**
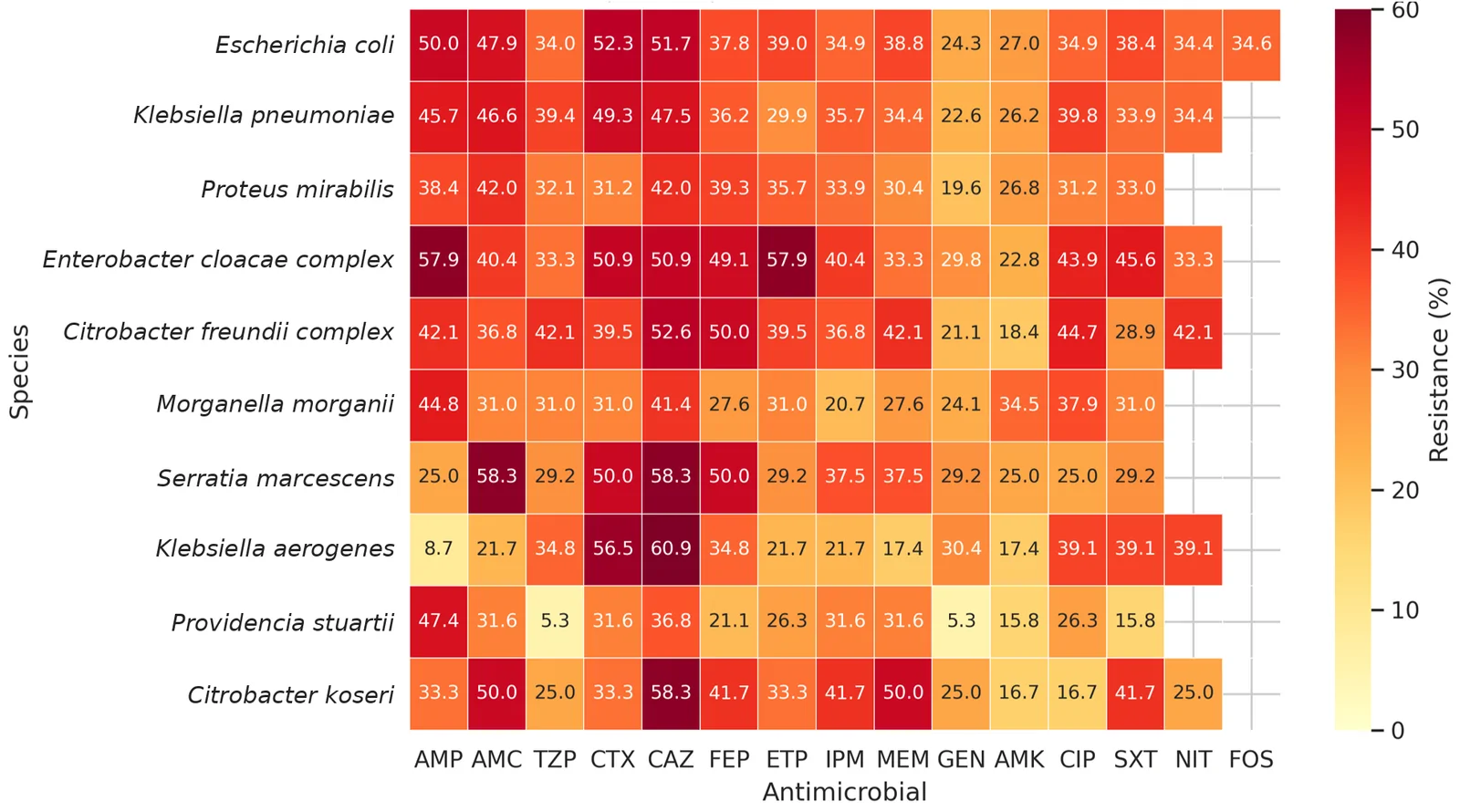
Species-specific resistance percentages for every recovered enterobacterales species and antimicrobial. Blank cells indicate combinations without an interpretable result. Antimicrobial abbreviations: AMP, ampicillin; AMC, amoxicillin-clavulanate; TZP, piperacillin-tazobactam; CTX, cefotaxime; CAZ, ceftazidime; FEP, cefepime; ETP, ertapenem; IPM, imipenem; MEM, meropenem; GEN, gentamicin; AMK, amikacin; CIP, ciprofloxacin; SXT, trimethoprim-sulfamethoxazole; NIT, nitrofurantoin; FOS, fosfomycin.

### 3.3 Biofilm, MDR, and phenotype combinations

Treatment failure across hospitals, biofilm intensity, MDR status, and primary species is summarized in Table 3 and illustrated in Figure 3. Biofilm production was common: 285/1,017 (28.0%) isolates were non-producers, 229/1,017 (22.5%) weak, 311/1,017 (30.6%) moderate, and 192/1,017 (18.9%) strong producers. At episode level, reconciled failure was 61/260 (23.5%) with no biofilm, 44/213 (20.7%) with weak, 50/301 (16.6%) with moderate, and 33/189 (17.5%) with strong maximum intensity (global *p* = 0.181). MDR-positive episodes had 94/544 failures (17.3%) compared with 94/419 (22.4%) among MDR-negative episodes (*p* = 0.055). These inverse crude patterns were treated as potentially confounded rather than protective.

**Table 3:** Treatment failure across hospitals, episode-level maximum biofilm intensity, MDR status, and primary species.

Comparison	Group	n	Failure *n* (%)	Global p
Hospital	H1	241	42 (17.4)	0.279
	H2	241	52 (21.6)	
	H3	241	54 (22.4)	
	H4	240	40 (16.7)	
Biofilm intensity	Non-producer	260	61 (23.5)	0.181
	Weak	213	44 (20.7)	
	Moderate	301	50 (16.6)	
	Strong	189	33 (17.5)	
MDR status	No	419	94 (22.4)	0.055
	Yes	544	94 (17.3)	
Primary species	*Citrobacter freundii complex*	38	8 (21.1)	0.241
	*Citrobacter koseri*	12	1 (8.3)	
	*Enterobacter cloacae complex*	55	10 (18.2)	
	*Escherichia coli*	455	79 (17.4)	
	*Klebsiella aerogenes*	21	4 (19.0)	
	*Klebsiella pneumoniae*	206	54 (26.2)	
	*Morganella morganii*	27	3 (11.1)	
	*Proteus mirabilis*	108	23 (21.3)	
	*Providencia stuartii*	18	4 (22.2)	
	*Serratia marcescens*	23	2 (8.7)	

**Figure 3:**
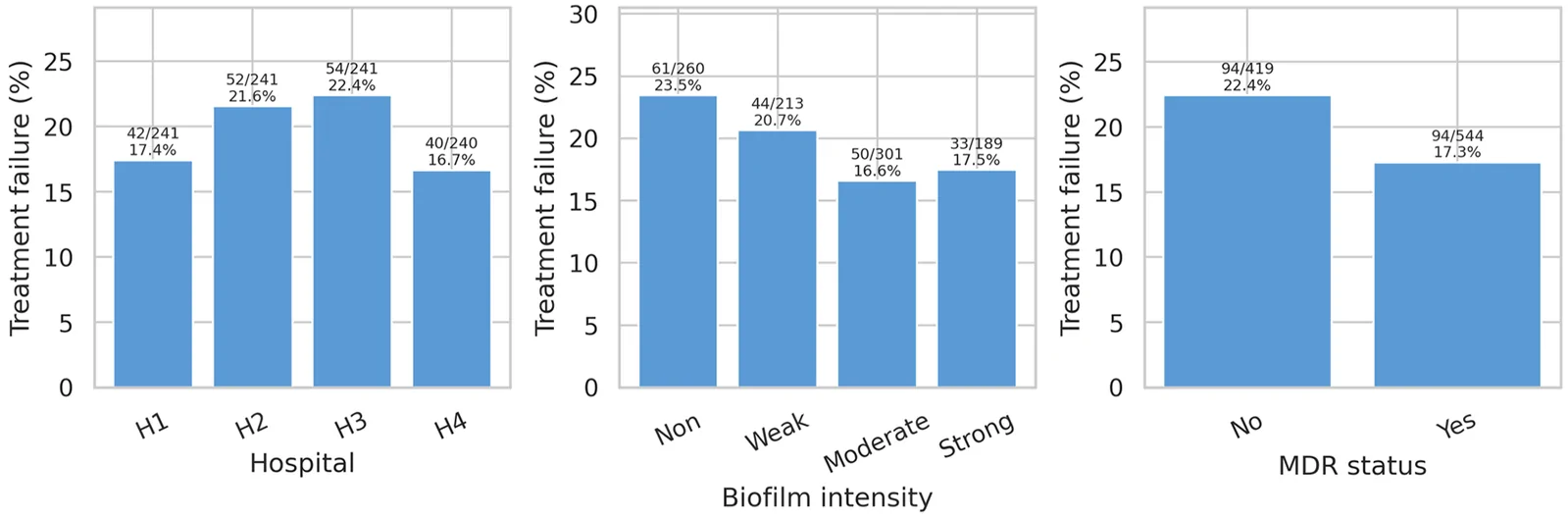
Reconciled treatment-failure proportions by hospital, episode-level maximum biofilm intensity, and MDR status.

Failure ranged from 16.7% to 22.4% across hospitals (*p* = 0.279). *K. pneumoniae* had the highest species-specific failure proportion (54/206, 26.2%), followed by *Providencia stuartii* (4/18, 22.2%) and *P. mirabilis* (23/108, 21.3%); the overall species comparison was not significant (*p* = 0.241).

### 3.4 Antimicrobial management and clinical course

Treatment and clinical-course characteristics according to outcome are summarized in Table 4. Empirical therapy was active *in vitro* in 695 episodes, inactive in 228, and indeterminate in 40. Source control was not required in 672 episodes, was required and adequate in 228, and was required but inadequate in 63. Because outcome components overlapped, their counts do not sum to the 188 failures. Across all 963 episodes, 113 (11.7%) had 72-hour clinical non-response, 80 (8.3%) had documented same-organism persistence at 3–7 days, 96 (10.0%) underwent antimicrobial escalation due to failure, 24 (2.5%) had ICU transfer after the index culture, 38 (3.9%) had recurrence within 30 days, 33 (3.4%) had UTI-related readmission within 30 days, and 7 (0.7%) died within 30 days; these descriptive component counts are detailed in Supplementary Table S4. UTI-specific mortality attribution was not reliably classifiable. A repeat urine culture at 3–7 days was available for 421/963 episodes (43.7%) and was not obtained for 542/963 (56.3%); among those cultured, 320 were negative, 101 were positive, and 80 of the 101 positive cultures recovered the same organism. Repeat culture was obtained in 140/188 failures (74.5%) versus 281/775 successes (36.3%; *p* < 0.001), demonstrating outcome-linked ascertainment. Repeat cultures were obtained for clinical indications rather than by a uniform surveillance schedule. The specific indication for each repeat culture was not captured as a structured variable, so indication-specific comparisons between outcome groups cannot be made. Baseline comparisons of cultured and non-cultured episodes and the *post hoc* persistence-deletion sensitivity analysis are provided in Supplementary Table S5.

**Table 4:** Treatment and clinical course according to treatment outcome.

Characteristic	Overall *n* = 963	Success *n* = 775	Failure *n* = 188
Empiric therapy active *in vitro*	695 (72.2)	576 (74.3)	119 (63.3)
Empiric therapy inactive *in vitro*	228 (23.7)	165 (21.3)	63 (33.5)
Empiric activity unknown	40 (4.2)	34 (4.4)	6 (3.2)
Time to active therapy hours	8.0 (5.0–30.0)	8.0 (5.0–26.0)	9.0 (5.0–44.0)
Source control not required	672 (69.8)	550 (71.0)	122 (64.9)
Source control required and adequate	228 (23.7)	181 (23.4)	47 (25.0)
Source control required but inadequate	63 (6.5)	44 (5.7)	19 (10.1)
Length of stay days	9.0 (6.0–12.0)	8.0 (5.0–12.0)	14.0 (10.0–21.0)

### 3.5 Multivariable analysis and model performance

In the prespecified primary explanatory model, obstruction, diabetes, and sepsis were associated with the reconciled treatment-failure outcome after adjustment. The full multivariable model is presented in Table 5. Phenotypic beta-lactamase co-production was not independently associated with failure, whereas biofilm positivity showed an inverse adjusted association (aOR 0.70, 95% CI 0.49–0.99; *p* = 0.045) that was not consistently statistically significant across sensitivity analyses. The principal adjusted associations are illustrated in Figure 4. No co-production-by-biofilm interaction was detected (aOR 0.75, 95% CI 0.24–2.36; *p* = 0.620). These estimates are interpreted as observational associations rather than causal or predictive effects.

**Table 5:** Prespecified multivariable logistic regression for treatment failure. Reference categories: male sex for sex and Hospital H1 for hospital fixed effects.

Predictor	aOR	95% CI	*p*
Beta lactamase co-production	0.62	0.35–1.09	0.097
Biofilm positive	0.70	0.49–0.99	0.045
Age per year	1.00	0.99–1.01	0.954
Female sex	1.17	0.83–1.65	0.374
Hospital H2 vs H1	1.38	0.87–2.19	0.173
Hospital H3 vs H1	1.42	0.90–2.26	0.133
Hospital H4 vs H1	0.93	0.57–1.51	0.758
Urinary catheter	1.00	0.71–1.42	0.993
Obstruction	1.55	1.06–2.26	0.025
Prior antibiotics 90 days	1.00	0.72–1.39	0.997
Diabetes	1.48	1.06–2.09	0.023
Chronic kidney disease	1.36	0.90–2.04	0.144
Charlson score per point	0.98	0.92–1.04	0.548
Sepsis	1.59	1.10–2.29	0.013
Polymicrobial infection	0.99	0.66–1.48	0.965

**Figure 4:**
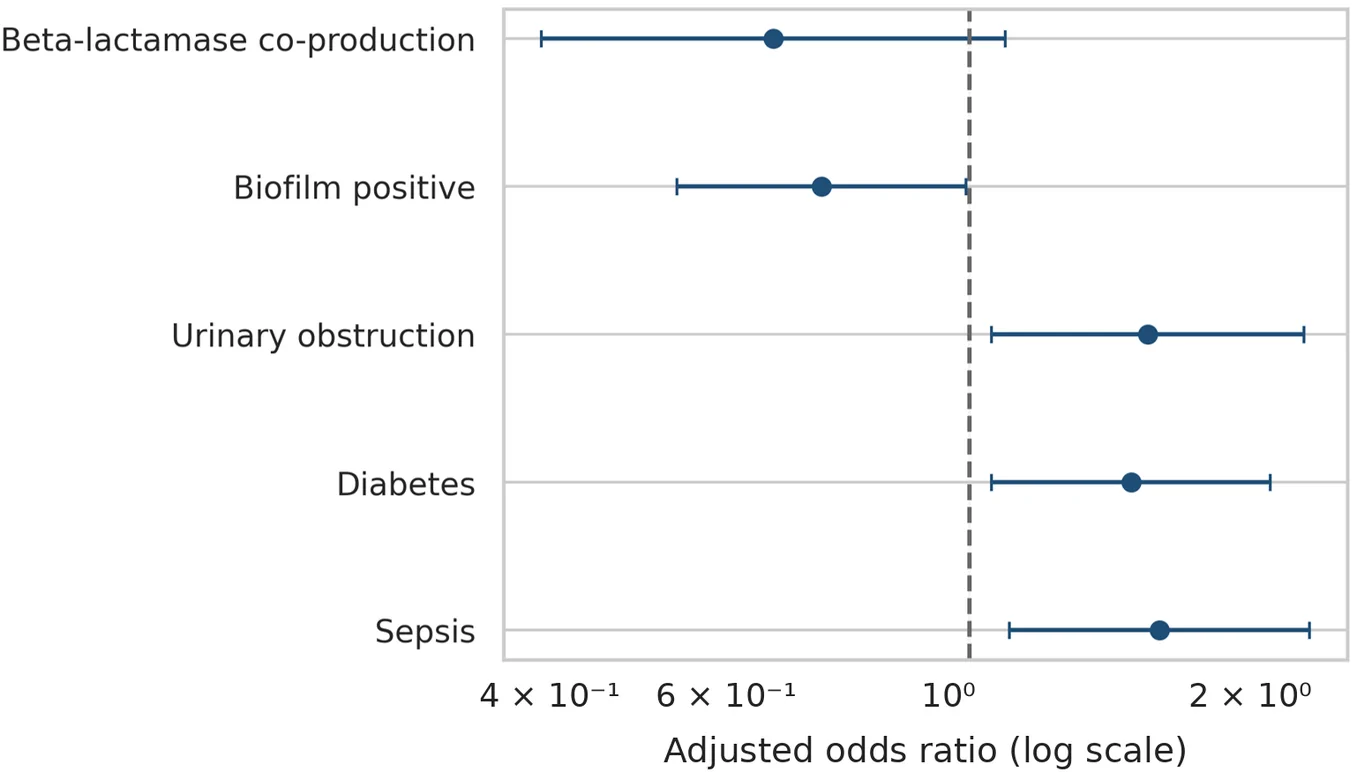
Adjusted associations of the principal microbiological exposures and clinical factors with reconciled treatment failure.

The primary model included 188 events and 15 non-intercept parameters. The apparent AUC was 0.623 (10,000-resample bootstrap 95% CI 0.608–0.690), whereas the optimism-corrected AUC was 0.575; the latter indicates limited discriminatory performance. The Brier score was 0.152. The apparent calibration intercept and slope were approximately 0 and 1, respectively, and the bootstrap-corrected calibration slope was 0.666. The Hosmer-Lemeshow statistic was 13.64 with 8 degrees of freedom (*p* = 0.092). Eighty-two observations exceeded the prespecified screening threshold of 4/n for approximate Cook distance; none was excluded from the analysis. These performance measures are reported as internal diagnostics of an explanatory model and should not be interpreted as validation of a bedside prediction tool. The receiver-operating-characteristic curve is shown in Figure 5, and the decile-based calibration plot is shown in Figure 6.

**Figure 5:**
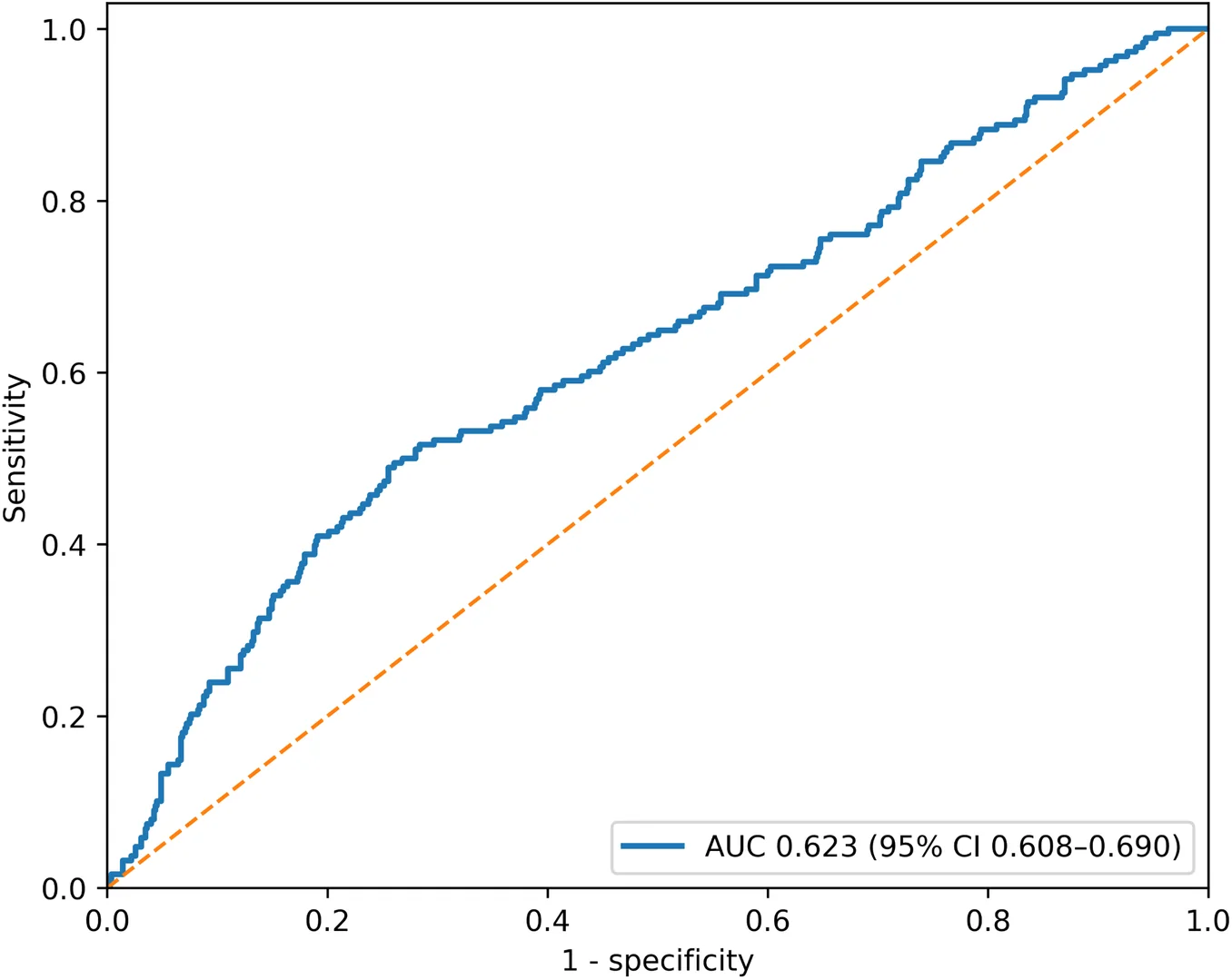
Receiver-operating-characteristic curve for the primary explanatory model. The plotted in-sample AUC is the apparent AUC (0.623); the optimism-corrected AUC from bootstrap internal validation was 0.575.

**Figure 6:**
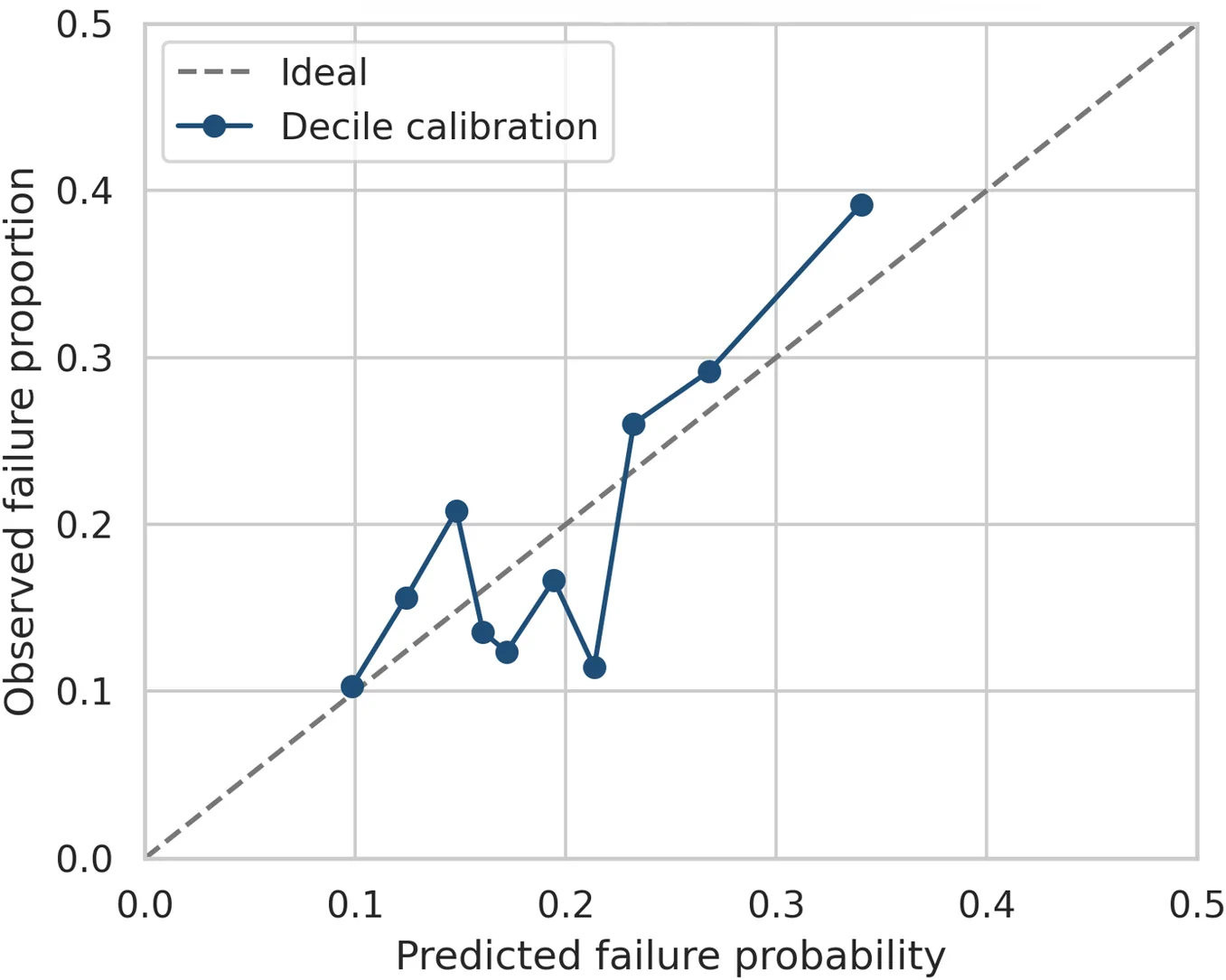
Decile-based calibration of predicted and observed treatment failure for all 963 episodes. Deciles 1−3 each contained 97 episodes and deciles 4−10 each contained 96 episodes. Points summarize groups formed from the distribution of predicted risk; the plot is an internal calibration diagnostic rather than external predictive validation.

The treatment-extended model included 923 episodes. Compared with episodes not requiring source control, required but inadequate source control was associated with higher odds of treatment failure (aOR 2.06, 95% CI 1.04–4.10; *p* = 0.039), whereas required and adequate source control was not significantly associated with failure (aOR 1.12, 95% CI 0.68–1.84; *p* = 0.666). Because source-control decisions were not randomized and are subject to confounding by indication, these estimates are associations and should not be interpreted as causal effects of source-control intervention. Empiric inactivity and time to active therapy were not independently associated with treatment failure. Sensitivity analyses for the two principal microbiological exposures are summarized in Table 6.

**Table 6:** Exploratory sensitivity analyses for the two principal microbiological exposures.

Analysis	n/events	Co-production aOR 95% CI	p	Biofilm aOR 95% CI	*p*
Primary reconciled model	963/188	0.62 (0.35–1.09)	0.097	0.70 (0.49–0.99)	0.045
Monomicrobial episodes only	762/149	0.67 (0.36–1.23)	0.195	0.76 (0.51–1.13)	0.168
Episodes with E. coli or K. pneumoniae	661/133	0.63 (0.33–1.22)	0.169	0.64 (0.42–0.97)	0.037
Excluding carbapenemase-positive episodes	856/170	0.69 (0.28–1.70)	0.416	0.66 (0.46–0.96)	0.029
Treatment-extended model	923/182	0.63 (0.35–1.14)	0.128	0.72 (0.50–1.03)	0.076
Firth penalized model	963/188	0.64 (0.37–1.11)	0.113	0.70 (0.49–0.99)	0.045
Original 152-event coding before outcome reconciliation	963/152	0.51 (0.26–0.98)	0.044	0.72 (0.49–1.06)	0.093
Composite excluding microbiological persistence (*post hoc*)	963/147	0.53 (0.27–1.03)	0.059	0.76 (0.51–1.13)	0.173

The persistence-deleted outcome was a *post hoc* sensitivity analysis; all models retain their stated covariate structures and are interpreted through effect estimates, confidence intervals, and consistency rather than isolated *p* values.

Across the exploratory sensitivity analyses, phenotypic beta-lactamase co-production was not significantly associated with treatment failure in the monomicrobial, E. coli/K. pneumoniae, carbapenemase-excluded, treatment-extended, or Firth penalized models (Table 6). In the model using the original 152-event coding, co-production showed an inverse association (aOR 0.51, 95% CI 0.26–0.98; *p* = 0.044), whereas biofilm positivity was not statistically significant (aOR 0.72, 95% CI 0.49–1.06; *p* = 0.093). In the *post hoc* analysis that omitted microbiological persistence from the composite, 147 episodes remained failures; neither co-production (aOR 0.53, 95% CI 0.27–1.03; *p* = 0.059) nor biofilm positivity (aOR 0.76, 95% CI 0.51–1.13; *p* = 0.173) was statistically significant. Obstruction, diabetes, and sepsis remained positively associated with failure in that analysis. Biofilm positivity showed inverse associations in the E. coli/K. pneumoniae subgroup and the carbapenemase-excluded and Firth models, but not in the monomicrobial, treatment-extended, original-coding, or persistence-deleted analyses. In the ordinal biofilm model, the aOR per increasing biofilm category was 0.84 (95% CI 0.72–0.98). Episode-level MDR was not independently associated with treatment failure (aOR 0.75, 95% CI 0.53–1.06). Given the number of secondary analyses and variation in statistical significance, these findings are interpreted by their effect estimates, confidence intervals, and cross-analysis consistency rather than by isolated *p* values.

## 4 Discussion

### 4.1 Principal findings

In this prospective four-hospital cohort of adults with Enterobacterales complicated urinary tract infection, host severity and remediable urinary-tract factors showed more consistent adjusted associations with treatment failure than the principal microbiological phenotypes evaluated. Urinary obstruction, diabetes, and sepsis were associated with failure after multivariable adjustment. Required but inadequate source control was also associated with higher odds of failure in the treatment-extended model, but this observational association is vulnerable to confounding by indication and is not a causal treatment-effect estimate. Phenotypic beta-lactamase co-production was not consistently associated with the reconciled outcome, while biofilm positivity showed an inverse association whose statistical significance varied across sensitivity analyses and therefore does not support a protective biological interpretation.

The cohort also demonstrated a substantial antimicrobial-resistance burden. More than one half of isolates met the MDR definition, more than one third produced ESBL, approximately one in six expressed an AmpC phenotype, and approximately one in ten were carbapenemase positive or co-production positive isolates. Biofilm formation was also common, occurring in nearly three quarters of isolates. These findings indicate that antimicrobial resistance and biofilm capacity were highly prevalent characteristics of the bacterial population, but prevalence alone did not translate directly into independent prognostic value for clinical failure.

An important feature of the analysis was the distinction between isolate-level microbiological characteristics and episode-level clinical outcome. A resistance phenotype can influence antimicrobial selection, but its effect on outcome may be modified by treatment adaptation, achievable urinary drug concentrations, underlying illness severity, urinary obstruction, catheter or device management, and source control. Similarly, *in-vitro* biofilm-forming capacity represents only one component of persistence and may not reproduce the dynamic environment of the urinary tract. The present findings therefore support a multidimensional interpretation of cUTI in which host, anatomical, therapeutic, and microbiological factors interact rather than act independently.

### 4.2 Host severity and urinary-tract determinants of treatment failure

Urinary obstruction emerged as one of the most consistent adverse factors in the primary model. This association is clinically plausible because obstruction can maintain bacterial burden, impair urinary drainage, increase intraluminal pressure, and reduce the likelihood that antimicrobial therapy alone will achieve clinical resolution. Contemporary cUTI frameworks increasingly emphasize that antimicrobial activity is only one component of treatment when an infected collecting system remains mechanically compromised (1, 2, 4, 5). In such circumstances, effective drainage or correction of the obstructing process may be required in parallel with antimicrobial therapy.

The persistence of the obstruction association after adjustment for demographic characteristics, comorbidities, sepsis, prior antimicrobial exposure, catheterization, hospital, and microbiological factors suggests that this finding was not simply a proxy for general illness severity. The association was also directionally consistent across clinically relevant sensitivity analyses. This is compatible with observations from severe pyelonephritis and obstructive urinary infection in which delayed drainage, shock, and major comorbidity have been associated with adverse outcomes (34, 35). Although the present observational analysis cannot establish a causal effect of drainage, the findings reinforce the clinical importance of identifying reversible urinary-tract abnormalities early in the course of cUTI.

Diabetes was also independently associated with treatment failure. Diabetes may contribute to adverse UTI outcomes through several mechanisms, including impaired innate and adaptive immune responses, glycosuria, autonomic bladder dysfunction, vascular disease, renal impairment, and increased cumulative exposure to healthcare environments and antimicrobials (36, 37). Importantly, the association remained after adjustment for age, chronic kidney disease, Charlson score, sepsis, and other measured covariates. This suggests that diabetes may contribute risk beyond its contribution to overall comorbidity burden.

The present finding is relevant because much of the literature on diabetes and UTI focuses on increased susceptibility to infection, recurrence, resistant organisms, or complicated presentation rather than treatment failure within an already-defined cUTI cohort. Our data extend that literature by demonstrating that diabetes retained an outcome association after restricting the study population to complicated infections. Nevertheless, the observational design does not allow separation of biological susceptibility from differences in treatment exposure, glycemic control, renal function, healthcare contact, or other unmeasured factors.

Sepsis was the third consistent independent clinical determinant. Patients presenting with sepsis have greater systemic inflammatory activation, a higher probability of organ dysfunction, and a narrower margin for delay in appropriate treatment and source control. The adjusted association observed here is therefore clinically coherent and underscores the importance of distinguishing bacterial phenotype from host response. An organism expressing a resistant phenotype may not necessarily result in treatment failure if effective therapy is rapidly instituted, whereas a susceptible isolate may still be associated with poor outcome in a patient with severe systemic illness, obstruction, or inadequate source control.

These findings can also be considered alongside the multicenter RESCUING study by Eliakim-Raz et al. (32), in which treatment failure occurred in 26.6% of 981 hospitalized patients with complicated urinary tract infection. In that cohort, adverse outcome was associated predominantly with markers of host vulnerability and illness severity, including intensive-care-unit admission, septic shock, bedridden status, metastatic cancer, and catheter-associated infection, whereas inappropriate empirical antimicrobial treatment and time to antibiotic initiation were not independently associated with treatment failure. Although the populations, outcome definitions, and analytical approaches differ, the present findings show a broadly similar pattern: sepsis and urinary-tract or host factors were more consistent correlates of failure than antimicrobial-management variables alone. The lower failure proportion in our cohort (19.5%) should therefore be interpreted cautiously rather than as evidence of a direct difference in prognosis between settings.

Taken together, these findings suggest that treatment failure in cUTI cannot be adequately explained by microbiological resistance alone. The host-pathogen relationship is strongly conditioned by severity of illness and by whether an anatomical or functional abnormality can be corrected.

### 4.3 Beta-lactamase phenotypes, co-production, and clinical outcome

The isolate collection showed a substantial burden of beta-lactamase-mediated resistance, with clear variation across Enterobacterales species. ESBL production was concentrated predominantly in *E. coli* and *K. pneumoniae*, whereas AmpC phenotypes were more prominent among species with established chromosomal AmpC potential, including *E. cloacae* complex, *C. freundii* complex, and *S. marcescens*. This species-level heterogeneity is clinically relevant because Enterobacterales differ in intrinsic resistance, inducible beta-lactamase biology, acquisition of plasmid-mediated determinants, and the antimicrobial agents for which susceptibility results remain clinically meaningful.

Species-stratified reporting therefore provides more useful information than a single pooled prevalence across all Enterobacterales. Similar heterogeneity has been described in studies from North Africa and other hospital settings, although direct comparisons are limited by differences in patient populations, organism distribution, phenotypic methods, antimicrobial exposure, and local resistance ecology (38–40).

Despite the high burden of resistance, beta-lactamase co-production did not emerge as a consistent independent predictor of treatment failure after adjustment for clinical factors. One plausible explanation is treatment adaptation. Recognition of broader resistance may influence antimicrobial selection, trigger escalation to more reliably active agents, prompt closer monitoring, or lead to earlier device management and source-control intervention. Such responses can weaken the apparent relationship between resistance phenotype and adverse outcome, particularly when resistant infections receive targeted escalation or alternative active therapy (41–45).

The clinical impact of a resistance phenotype also depends on the treatment context in which it occurs. Empirical activity, subsequent modification of therapy, infection severity, renal function, achievable urinary drug concentrations, and adequacy of source control may all modify the effect of resistance on outcome. Current AMR guidance similarly distinguishes ESBL-, AmpC-, and carbapenemase-producing Enterobacterales therapeutically rather than assuming that all resistance mechanisms confer the same prognostic effect (6–9, 41–45).

Phenotypic beta-lactamase co-production is an operational laboratory category rather than a single molecular mechanism. The same phenotypic combination may arise from different enzymes, regulatory mechanisms, or overlapping resistance determinants. Because molecular confirmation was not performed, the present exposure cannot establish genetic co-carriage of ESBL, AmpC, carbapenemase, or other resistance genes. The null primary association should therefore be interpreted as applying to the composite phenotypic definition used here; phenotype misclassification and mechanistic heterogeneity could attenuate or obscure an association with specific genetic resistance mechanisms.

The sensitivity analyses reinforce this caution. The direction and statistical strength of the co-production estimate changed with outcome coding, including an inverse estimate in the original 152-event coding; when microbiological persistence was removed from the composite, the estimate was attenuated and its confidence interval crossed unity (aOR 0.53, 95% CI 0.27–1.03). This instability, together with differential verification of microbiological persistence, argues against interpreting either the primary null result or isolated inverse estimates as evidence about a specific biological mechanism.

Accordingly, the data do not show that beta-lactamase co-production is clinically unimportant; rather, the prespecified phenotypic exposure did not function as a robust independent prognostic marker of treatment failure in this cohort. Its major clinical relevance remains antimicrobial susceptibility interpretation and treatment selection.

### 4.4 Biofilm formation and the inverse observational association

Biofilm formation was common, but the inverse adjusted association with treatment failure was of limited robustness and should not be interpreted as evidence of biological protection. The microtiter-plate assay measures attached biomass under standardized static conditions, whereas *in-vivo* persistence is influenced by urinary flow, host immunity, antimicrobial exposure, epithelial and device surfaces, and mixed-species interactions. Thus, an *in-vitro* biofilm phenotype and patient-level prognosis are related but non-equivalent constructs.

The observational estimate may also reflect species composition, treatment adaptation, device management, source control, residual confounding, and differential follow-up culture. Because repeat cultures were obtained for clinical indications, the opportunity to detect microbiological persistence was unequal across participants, directly linking outcome verification to one of the processes biofilm might influence.

Most importantly, the biofilm association was not consistently statistically significant in the monomicrobial and treatment-extended analyses, and it was also non-significant when microbiological persistence was removed from the composite (aOR 0.76, 95% CI 0.51–1.13). Its significance therefore depended on outcome definition and analytic subset. We regard the finding as an epidemiological association requiring external confirmation, not as evidence that biofilm reduces failure risk. The result does not challenge the established biological role of biofilm in persistence or device-associated infection.

### 4.5 Antimicrobial resistance patterns and therapeutic implications

The antimicrobial susceptibility profile illustrates the substantial resistance burden among urinary Enterobacterales in this cohort and reinforces the need for locally generated susceptibility data. Similar concerns have been reported across African and international settings, where resistance patterns vary markedly according to geography, hospital environment, patient population, antimicrobial exposure, and species composition (10–15, 46).

Species-specific interpretation is particularly important because pooled Enterobacterales susceptibility estimates can conceal clinically meaningful differences. *E. coli* and *K. pneumoniae* contributed much of the cephalosporin resistance burden, whereas other Enterobacterales displayed distinct beta-lactam resistance patterns reflecting their underlying species-specific resistance biology. This heterogeneity limits the value of relying on a single pooled resistance estimate when making organism-specific therapeutic decisions.

The same principle applies to agents whose interpretation depends on species or clinical indication. Restricting susceptibility denominators to clinically interpretable organism-drug combinations avoids misleading pooled estimates and provides a more accurate representation of the local antibiogram. This is especially relevant for urinary agents such as nitrofurantoin and fosfomycin, for which susceptibility results should not be generalized indiscriminately across all Enterobacterales.

The MIC analysis should also be interpreted within the context of its sampling design. Because testing was enriched for isolates with clinically important resistance phenotypes, the resulting MIC distributions characterize selected resistant subgroups rather than the entire isolate population. Their principal value is therefore phenotypic characterization of critical resistance, not estimation of population-wide MIC distributions.

The absence of a consistent independent association between episode-level MDR and treatment failure does not diminish the clinical importance of multidrug resistance. MDR directly affects empirical treatment options, the probability of initial antimicrobial inactivity, and the complexity of definitive therapy. However, its prognostic effect may be modified substantially by recognition of resistance, subsequent treatment adjustment, achievable urinary drug concentrations, host severity, anatomical abnormalities, and source-control measures. In this context, resistance phenotype is better viewed as one component of a broader clinical pathway rather than as a standalone determinant of outcome.

These findings support the use of species-resolved susceptibility reporting and emphasize that therapeutic decisions should integrate microbiological data with clinical severity, infection site, host characteristics, and source-control requirements. Local resistance surveillance remains essential, but susceptibility results are most informative when interpreted within the broader clinical context of the individual cUTI episode.

### 4.6 Antimicrobial management, source control, and treatment adaptation

Source control emerged as an important clinical dimension of treatment outcome. The key distinction was not simply whether a source-control problem was present, but whether that problem was adequately addressed. This is clinically relevant because complicated urinary infection often occurs in the setting of obstruction, indwelling devices, impaired drainage, or other anatomical abnormalities that may sustain infection despite appropriate antimicrobial therapy.

Adequate source control can modify the clinical consequences of these abnormalities by reducing bacterial burden and removing or correcting a persistent focus of infection. Conversely, antimicrobial therapy alone may be insufficient when obstruction remains unresolved, an infected device is retained, or drainage is inadequate. This interpretation is consistent with current management principles for obstructive and device-associated urinary infection, in which correction or removal of the underlying source forms an essential component of treatment (47–49).

The observational nature of the study, however, requires caution. Decisions regarding drainage, catheter replacement, device removal, or other interventions were made as part of routine clinical care rather than assigned experimentally. Source-control status may therefore reflect both disease severity and clinician judgment, and the present findings should not be interpreted as a causal estimate of the effect of intervention.

The antimicrobial-management findings should be interpreted with similar caution. The absence of an independent association for empirical *in-vitro* inactivity or time to active therapy does not imply that antimicrobial activity or timely treatment are clinically unimportant. Treatment variables are highly susceptible to confounding by indication because patients with more severe disease or more resistant organisms may receive broader initial therapy, earlier escalation, or more intensive monitoring. Such treatment adaptation can obscure simple associations between antimicrobial exposure and outcome (50–53).

Time to active therapy is also difficult to represent fully using a single linear measure. The clinical effect of delay may vary according to severity, infection source, renal function, bacterial susceptibility, and the timing of source control. In addition, antimicrobial treatment frequently changes after microbiological results become available, making baseline treatment variables an incomplete representation of the therapeutic course (50–53).

These findings therefore support an integrated approach to cUTI management in which antimicrobial susceptibility is interpreted alongside host severity, anatomical abnormalities, device status, and adequacy of source control. Resistance remains clinically important, but successful treatment depends on more than antimicrobial activity alone.

### 4.7 Outcome ascertainment, repeat cultures, and verification bias

Outcome ascertainment is a major limitation because follow-up urine cultures were obtained according to clinical indication rather than a uniform surveillance protocol. Consequently, only 421/963 episodes (43.7%) had repeat cultures, and patients selected for repeat culture were not a random sample of the cohort. Repeat culture was obtained in 74.5% of reconciled failures versus 36.3% of successes. The specific clinical indication for each culture was not recorded as a structured variable, so indication-specific differences between outcome groups cannot be quantified.

This creates differential verification: the opportunity to detect same-organism persistence was unequal across participants. Because persistence was one component of the composite outcome and the principal exposures included biofilm and resistance phenotypes, this bias could influence both the observed failure frequency and microbiological effect estimates.

The cultured-versus-non-cultured comparison in Supplementary Table S5 characterizes measured baseline differences but cannot eliminate unmeasured selection into follow-up culture. The analysis using the original 152-event coding assessed sensitivity to correction of the 36 discordant records. Separately, the *post hoc* persistence-deletion sensitivity analysis addressed a different question by removing microbiological persistence from the composite outcome entirely. This left 147 failures, and neither co-production nor biofilm positivity was statistically significant. The attenuation of the biofilm association in this analysis indicates that the inverse association observed in the primary model was sensitive to outcome definition. However, deleting the persistence component cannot eliminate verification bias because unrecognized persistence may still have occurred among participants without repeat culture and the decision to obtain a repeat culture remained clinically selected.

The change in microbiological estimates across outcome definitions shows that some associations are sensitive to outcome ascertainment. Microbiological persistence should therefore be regarded as incompletely verified in the full cohort, and inverse biofilm or co-production estimates should not be interpreted as stable biological effects.

Future studies should use standardized follow-up assessments and, when clinically appropriate, scheduled cultures so that clinical response and microbiological persistence can be analyzed separately as well as within a composite endpoint.

### 4.8 Strengths and limitations

This study has several strengths. Its prospective multicenter design, separation of episode-level and isolate-level analyses, inclusion of polymicrobial infections, and preservation of raw inhibition-zone measurements provided a strong framework for linking clinical outcomes with microbiological characteristics. Retaining the original zone diameters also allowed final susceptibility categories to be harmonized using the most recent CLSI breakpoints without implying that the 2026 interpretive edition had been applied prospectively throughout the study period.

Microbiological characterization extended beyond routine identification and disk diffusion to include phenotypic ESBL, AmpC, carbapenemase, and co-production testing, selected agar-dilution MIC measurements, and triplicate microtiter-plate biofilm assessment. Independent phenotype reading, quality-control procedures, species-specific susceptibility denominators, explicit handling of not-applicable results, and extensive supplementary reporting further strengthened reproducibility and transparency.

The analytical approach was also strengthened by the use of prespecified clinically relevant covariates rather than automated variable selection, together with multiple sensitivity analyses addressing organism composition, polymicrobial infection, carbapenemase status, penalized estimation, biofilm intensity, MDR, treatment variables, and alternative outcome coding. Bootstrap internal validation provided an additional assessment of model optimism and calibration.

Several limitations should nevertheless be considered. The observational design prevents causal inference and leaves the findings vulnerable to residual confounding and confounding by indication, particularly for antimicrobial management and source-control interventions. The treatment-failure endpoint intentionally combined clinically important but heterogeneous events, including early non-response, microbiological persistence, escalation, deterioration, recurrence, readmission, and death. These components can have different determinants and can overlap; component-level descriptive reporting is therefore complementary to the composite analysis.

Microbiological persistence was not verified uniformly because repeat urine cultures were clinically indicated rather than protocol-mandated. Repeat culture was substantially more frequent among failures than successes, confirming outcome-linked ascertainment. Although baseline cultured-versus-non-cultured comparisons and a *post hoc* sensitivity analysis excluding microbiological persistence from the composite were performed, unrecognized persistence among patients without repeat culture cannot be excluded. Differential verification may therefore have influenced associations involving biofilm and resistance phenotypes.

Beta-lactamase characterization was phenotypic rather than molecular. The study could not identify specific ESBL, AmpC, or carbapenemase genes, enzyme variants, plasmid-mediated mechanisms, or clonal relationships; phenotypic overlap may also have affected the co-production classification. Co-production should therefore be understood as the prespecified phenotype of two or more detected beta-lactamase categories, not as demonstrated genetic co-carriage.

Biofilm assessment used three technical replicate wells rather than independent biological replication across separate experiments. The assay measures static crystal-violet biomass and does not reproduce the full urinary-tract environment; no formal inter-run reproducibility statistic was prespecified. In polymicrobial episodes, the any-isolate exposure rule could also misclassify the episode-level contribution of a phenotype because it does not identify the organism primarily responsible for the clinical course; the monomicrobial sensitivity analysis partly addresses this concern.

Species identification was strengthened by conventional testing, API 20E, and MALDI confirmation, but the specific MALDI instrument model and software/database version identifiers were not retained in the analytic dataset. These details could not be reconstructed retrospectively and should therefore be considered a minor reproducibility limitation.

The regression model showed limited discriminatory performance after optimism correction (AUC 0.575) and some optimism in calibration. It was designed to estimate adjusted associations, not to provide individualized clinical prediction. Events per parameter are reported descriptively, while the prespecified covariate structure and bootstrap internal validation provide the more relevant context for model stability.

Finally, all participating hospitals were located within one city. Although the cohort included both public and private institutions and contributes data from a setting that is underrepresented in the cUTI literature, local microbiological ecology, antimicrobial use, referral patterns, and healthcare structure may limit generalizability to other regions.

### 4.9 Clinical and research implications

The findings support an integrated approach to cUTI risk assessment. Antimicrobial susceptibility remains essential for treatment selection, but clinical severity, urinary obstruction, diabetes, sepsis, device-related factors, and adequacy of source control appear to provide complementary information about the risk of treatment failure. Microbiological phenotype should therefore be interpreted within the broader clinical context rather than used in isolation.

Phenotypic beta-lactamase co-production and *in-vitro* biofilm formation should not be regarded as standalone prognostic biomarkers on the basis of the present data. Co-production remains clinically important because it can restrict antimicrobial options and complicate susceptibility interpretation, while biofilm remains biologically relevant to persistence and device-associated infection. Their prognostic value, however, appears to be strongly influenced by host factors, treatment adaptation, species distribution, measurement limitations, and outcome ascertainment. Previous evidence from Misurata has similarly shown that ESBL/AmpC co-production in uropathogenic *Escherichia coli* is associated with a substantial multidrug-resistance burden and is particularly prevalent among patients with diabetes, supporting the importance of interpreting resistance phenotypes together with relevant host factors (54).

Future studies should combine detailed microbiological characterization with more standardized longitudinal clinical follow-up. Uniform outcome assessment and scheduled follow-up cultures, when clinically appropriate, would reduce differential verification. More detailed time-varying treatment data could improve evaluation of antimicrobial adaptation, while molecular characterization would help distinguish specific resistance genes, plasmid-mediated mechanisms, and clonal lineages.

Biofilm research would also benefit from biological replication, clinically relevant surfaces, device-based models, and experimental conditions that better approximate the urinary environment. Evaluating biofilm intensity alongside clinical and molecular characteristics may provide more informative insight than relying on a simple positive-negative classification.

Validation in other Libyan regions and in geographically distinct healthcare systems would help determine the reproducibility and transportability of the observed associations. Such studies could also clarify the extent to which local resistance ecology modifies the relationship between microbiological phenotype and clinical outcome.

## 5 Conclusion

In this cohort, treatment failure in complicated urinary tract infection was associated more consistently with host severity, urinary-tract abnormalities, and treatment-context factors than with the microbiological phenotypes evaluated. Phenotypic beta-lactamase co-production was not a robust independent prognostic marker. Biofilm positivity showed an inverse association in the primary model, but this finding was not consistent across sensitivity analyses and was attenuated in the *post hoc* analysis excluding microbiological persistence from the composite outcome; it should therefore not be interpreted as biologically protective. These observational findings should be considered in light of the heterogeneous composite endpoint, differential verification of microbiological persistence, phenotypic rather than molecular resistance characterization, technical rather than biological biofilm replication, and limited model discrimination. Species-resolved susceptibility reporting and assessment of remediable urinary-tract abnormalities remain clinically relevant, while future studies should incorporate standardized follow-up, component-specific outcomes, biological replication for biofilm assays, and molecular characterization.

## Data Availability

The original contributions presented in the study are included in the article/Supplementary Material, further inquiries can be directed to the corresponding author.
