# Supplementary material for: Clinical and microbiological determinants of treatment failure in complicated urinary tract infection: a four-hospital cohort of enterobacterales infections in Misurata, Libya
Source: Front Med (Lausanne). 2026 Oct 19;13:2002811. doi: 10.3389/fmed.2026.2002811 (PMC13724622; doi:10.3389/fmed.2026.2002811)
Supplement: Supplementary file 1 [file Datasheet1.docx]

S1. Exact isolate-selection rules and counts for agar-dilution MIC testing; final MIC categories were harmonized to CLSI M100 Ed36 (2026).

| Antibiotic | Selection rule | Reason for MIC | Isolates n |
| --- | --- | --- | --- |
| Cefepime | All 107 carbapenemase-positive isolates only | Critical-phenotype MIC characterization | 107 |
| Meropenem | Additional carbapenemase-negative isolates selected ... according to the laboratory MIC sampling plan, without selection on treatment outcome | Critical-phenotype characterization plus additional carbapenemase-negative comparison isolates | 224 |

S2. Confirmed ESBL, AmpC, and carbapenemase phenotype combinations.

| Phenotype pattern | n | Percent |
| --- | --- | --- |
| None confirmed | 482 | 47.4 |
| ESBL | 275 | 27.0 |
| AmpC | 115 | 11.3 |
| ESBL+Carbapenemase | 47 | 4.6 |
| ESBL+AmpC | 38 | 3.7 |
| Carbapenemase | 33 | 3.2 |
| AmpC+Carbapenemase | 15 | 1.5 |
| ESBL+AmpC+Carbapenemase | 12 | 1.2 |

S3. Complete species-specific antibiogram with tested denominators and S, I/SDD, R, and not-applicable counts.

| Species | Antimicrobial | Tested n | S n | S % | I (SDD) n | I (SDD) % | R n | R % | Not applicable n |
| --- | --- | --- | --- | --- | --- | --- | --- | --- | --- |
| Escherichia coli | AMP | 482 | 198 | 41.1 | 43 | 8.9 | 241 | 50.0 | 0 |
|  | AMC | 482 | 212 | 44.0 | 39 | 8.1 | 231 | 47.9 | 0 |
|  | TZP | 482 | 291 | 60.4 | 27 | 5.6 | 164 | 34.0 | 0 |
|  | CTX | 482 | 190 | 39.4 | 40 | 8.3 | 252 | 52.3 | 0 |
|  | CAZ | 482 | 202 | 41.9 | 31 | 6.4 | 249 | 51.7 | 0 |
|  | FEP | 482 | 255 | 52.9 | 45 | 9.3 | 182 | 37.8 | 0 |
|  | ETP | 482 | 256 | 53.1 | 38 | 7.9 | 188 | 39.0 | 0 |
|  | IPM | 482 | 276 | 57.3 | 38 | 7.9 | 168 | 34.9 | 0 |
|  | MEM | 482 | 266 | 55.2 | 29 | 6.0 | 187 | 38.8 | 0 |
|  | GEN | 482 | 317 | 65.8 | 48 | 10.0 | 117 | 24.3 | 0 |
|  | AMK | 482 | 305 | 63.3 | 47 | 9.8 | 130 | 27.0 | 0 |
|  | CIP | 482 | 266 | 55.2 | 48 | 10.0 | 168 | 34.9 | 0 |
|  | SXT | 482 | 257 | 53.3 | 40 | 8.3 | 185 | 38.4 | 0 |
|  | NIT | 482 | 274 | 56.8 | 42 | 8.7 | 166 | 34.4 | 0 |
|  | FOS | 482 | 276 | 57.3 | 39 | 8.1 | 167 | 34.6 | 0 |
| Proteus mirabilis | AMP | 112 | 60 | 53.6 | 9 | 8.0 | 43 | 38.4 | 0 |
|  | AMC | 112 | 57 | 50.9 | 8 | 7.1 | 47 | 42.0 | 0 |
|  | TZP | 112 | 70 | 62.5 | 6 | 5.4 | 36 | 32.1 | 0 |
|  | CTX | 112 | 69 | 61.6 | 8 | 7.1 | 35 | 31.3 | 0 |
|  | CAZ | 112 | 56 | 50.0 | 9 | 8.0 | 47 | 42.0 | 0 |
|  | FEP | 112 | 61 | 54.5 | 7 | 6.3 | 44 | 39.3 | 0 |
|  | ETP | 112 | 65 | 58.0 | 7 | 6.3 | 40 | 35.7 | 0 |
|  | IPM | 112 | 65 | 58.0 | 9 | 8.0 | 38 | 33.9 | 0 |
|  | MEM | 112 | 72 | 64.3 | 6 | 5.4 | 34 | 30.4 | 0 |
|  | GEN | 112 | 81 | 72.3 | 9 | 8.0 | 22 | 19.6 | 0 |
|  | AMK | 112 | 77 | 68.8 | 5 | 4.5 | 30 | 26.8 | 0 |
|  | CIP | 112 | 74 | 66.1 | 3 | 2.7 | 35 | 31.3 | 0 |
|  | SXT | 112 | 67 | 59.8 | 8 | 7.1 | 37 | 33.0 | 0 |
|  | NIT | 0 | 0 |  | 0 |  | 0 |  | 112 |
|  | FOS | 0 | 0 |  | 0 |  | 0 |  | 112 |
| Klebsiella aerogenes | AMP | 23 | 20 | 87.0 | 1 | 4.3 | 2 | 8.7 | 0 |
|  | AMC | 23 | 17 | 73.9 | 1 | 4.3 | 5 | 21.7 | 0 |
|  | TZP | 23 | 11 | 47.8 | 4 | 17.4 | 8 | 34.8 | 0 |
|  | CTX | 23 | 9 | 39.1 | 1 | 4.3 | 13 | 56.5 | 0 |
|  | CAZ | 23 | 6 | 26.1 | 3 | 13.0 | 14 | 60.9 | 0 |
|  | FEP | 23 | 14 | 60.9 | 1 | 4.3 | 8 | 34.8 | 0 |
|  | ETP | 23 | 15 | 65.2 | 3 | 13.0 | 5 | 21.7 | 0 |
|  | IPM | 23 | 16 | 69.6 | 2 | 8.7 | 5 | 21.7 | 0 |
|  | MEM | 23 | 19 | 82.6 | 0 | 0.0 | 4 | 17.4 | 0 |
|  | GEN | 23 | 15 | 65.2 | 1 | 4.3 | 7 | 30.4 | 0 |
|  | AMK | 23 | 17 | 73.9 | 2 | 8.7 | 4 | 17.4 | 0 |
|  | CIP | 23 | 12 | 52.2 | 2 | 8.7 | 9 | 39.1 | 0 |
|  | SXT | 23 | 13 | 56.5 | 1 | 4.3 | 9 | 39.1 | 0 |
|  | NIT | 23 | 13 | 56.5 | 1 | 4.3 | 9 | 39.1 | 0 |
|  | FOS | 0 | 0 |  | 0 |  | 0 |  | 23 |
| Klebsiella pneumoniae | AMP | 221 | 97 | 43.9 | 23 | 10.4 | 101 | 45.7 | 0 |
|  | AMC | 221 | 96 | 43.4 | 22 | 10.0 | 103 | 46.6 | 0 |
|  | TZP | 221 | 117 | 52.9 | 17 | 7.7 | 87 | 39.4 | 0 |
|  | CTX | 221 | 98 | 44.3 | 14 | 6.3 | 109 | 49.3 | 0 |
|  | CAZ | 221 | 98 | 44.3 | 18 | 8.1 | 105 | 47.5 | 0 |
|  | FEP | 221 | 123 | 55.7 | 18 | 8.1 | 80 | 36.2 | 0 |
|  | ETP | 221 | 135 | 61.1 | 20 | 9.0 | 66 | 29.9 | 0 |
|  | IPM | 221 | 126 | 57.0 | 16 | 7.2 | 79 | 35.7 | 0 |
|  | MEM | 221 | 133 | 60.2 | 12 | 5.4 | 76 | 34.4 | 0 |
|  | GEN | 221 | 155 | 70.1 | 16 | 7.2 | 50 | 22.6 | 0 |
|  | AMK | 221 | 149 | 67.4 | 14 | 6.3 | 58 | 26.2 | 0 |
|  | CIP | 221 | 116 | 52.5 | 17 | 7.7 | 88 | 39.8 | 0 |
|  | SXT | 221 | 132 | 59.7 | 14 | 6.3 | 75 | 33.9 | 0 |
|  | NIT | 221 | 130 | 58.8 | 15 | 6.8 | 76 | 34.4 | 0 |
|  | FOS | 0 | 0 |  | 0 |  | 0 |  | 221 |
| Enterobacter cloacae complex | AMP | 57 | 17 | 29.8 | 7 | 12.3 | 33 | 57.9 | 0 |
|  | AMC | 57 | 31 | 54.4 | 3 | 5.3 | 23 | 40.4 | 0 |
|  | TZP | 57 | 34 | 59.6 | 4 | 7.0 | 19 | 33.3 | 0 |
|  | CTX | 57 | 19 | 33.3 | 9 | 15.8 | 29 | 50.9 | 0 |
|  | CAZ | 57 | 26 | 45.6 | 2 | 3.5 | 29 | 50.9 | 0 |
|  | FEP | 57 | 26 | 45.6 | 3 | 5.3 | 28 | 49.1 | 0 |
|  | ETP | 57 | 19 | 33.3 | 5 | 8.8 | 33 | 57.9 | 0 |
|  | IPM | 57 | 30 | 52.6 | 4 | 7.0 | 23 | 40.4 | 0 |
|  | MEM | 57 | 28 | 49.1 | 10 | 17.5 | 19 | 33.3 | 0 |
|  | GEN | 57 | 38 | 66.7 | 2 | 3.5 | 17 | 29.8 | 0 |
|  | AMK | 57 | 40 | 70.2 | 4 | 7.0 | 13 | 22.8 | 0 |
|  | CIP | 57 | 25 | 43.9 | 7 | 12.3 | 25 | 43.9 | 0 |
|  | SXT | 57 | 28 | 49.1 | 3 | 5.3 | 26 | 45.6 | 0 |
|  | NIT | 57 | 33 | 57.9 | 5 | 8.8 | 19 | 33.3 | 0 |
|  | FOS | 0 | 0 |  | 0 |  | 0 |  | 57 |
| Morganella morganii | AMP | 29 | 14 | 48.3 | 2 | 6.9 | 13 | 44.8 | 0 |
|  | AMC | 29 | 15 | 51.7 | 5 | 17.2 | 9 | 31.0 | 0 |
|  | TZP | 29 | 19 | 65.5 | 1 | 3.4 | 9 | 31.0 | 0 |
|  | CTX | 29 | 19 | 65.5 | 1 | 3.4 | 9 | 31.0 | 0 |
|  | CAZ | 29 | 16 | 55.2 | 1 | 3.4 | 12 | 41.4 | 0 |
|  | FEP | 29 | 18 | 62.1 | 3 | 10.3 | 8 | 27.6 | 0 |
|  | ETP | 29 | 19 | 65.5 | 1 | 3.4 | 9 | 31.0 | 0 |
|  | IPM | 29 | 21 | 72.4 | 2 | 6.9 | 6 | 20.7 | 0 |
|  | MEM | 29 | 21 | 72.4 | 0 | 0.0 | 8 | 27.6 | 0 |
|  | GEN | 29 | 20 | 69.0 | 2 | 6.9 | 7 | 24.1 | 0 |
|  | AMK | 29 | 18 | 62.1 | 1 | 3.4 | 10 | 34.5 | 0 |
|  | CIP | 29 | 15 | 51.7 | 3 | 10.3 | 11 | 37.9 | 0 |
|  | SXT | 29 | 18 | 62.1 | 2 | 6.9 | 9 | 31.0 | 0 |
|  | NIT | 0 | 0 |  | 0 |  | 0 |  | 29 |
|  | FOS | 0 | 0 |  | 0 |  | 0 |  | 29 |
| Providencia stuartii | AMP | 19 | 9 | 47.4 | 1 | 5.3 | 9 | 47.4 | 0 |
|  | AMC | 19 | 9 | 47.4 | 4 | 21.1 | 6 | 31.6 | 0 |
|  | TZP | 19 | 16 | 84.2 | 2 | 10.5 | 1 | 5.3 | 0 |
|  | CTX | 19 | 11 | 57.9 | 2 | 10.5 | 6 | 31.6 | 0 |
|  | CAZ | 19 | 11 | 57.9 | 1 | 5.3 | 7 | 36.8 | 0 |
|  | FEP | 19 | 12 | 63.2 | 3 | 15.8 | 4 | 21.1 | 0 |
|  | ETP | 19 | 13 | 68.4 | 1 | 5.3 | 5 | 26.3 | 0 |
|  | IPM | 19 | 12 | 63.2 | 1 | 5.3 | 6 | 31.6 | 0 |
|  | MEM | 19 | 12 | 63.2 | 1 | 5.3 | 6 | 31.6 | 0 |
|  | GEN | 19 | 16 | 84.2 | 2 | 10.5 | 1 | 5.3 | 0 |
|  | AMK | 19 | 15 | 78.9 | 1 | 5.3 | 3 | 15.8 | 0 |
|  | CIP | 19 | 12 | 63.2 | 2 | 10.5 | 5 | 26.3 | 0 |
|  | SXT | 19 | 13 | 68.4 | 3 | 15.8 | 3 | 15.8 | 0 |
|  | NIT | 0 | 0 |  | 0 |  | 0 |  | 19 |
|  | FOS | 0 | 0 |  | 0 |  | 0 |  | 19 |
| Citrobacter koseri | AMP | 12 | 8 | 66.7 | 0 | 0.0 | 4 | 33.3 | 0 |
|  | AMC | 12 | 6 | 50.0 | 0 | 0.0 | 6 | 50.0 | 0 |
|  | TZP | 12 | 9 | 75.0 | 0 | 0.0 | 3 | 25.0 | 0 |
|  | CTX | 12 | 6 | 50.0 | 2 | 16.7 | 4 | 33.3 | 0 |
|  | CAZ | 12 | 5 | 41.7 | 0 | 0.0 | 7 | 58.3 | 0 |
|  | FEP | 12 | 7 | 58.3 | 0 | 0.0 | 5 | 41.7 | 0 |
|  | ETP | 12 | 8 | 66.7 | 0 | 0.0 | 4 | 33.3 | 0 |
|  | IPM | 12 | 5 | 41.7 | 2 | 16.7 | 5 | 41.7 | 0 |
|  | MEM | 12 | 5 | 41.7 | 1 | 8.3 | 6 | 50.0 | 0 |
|  | GEN | 12 | 8 | 66.7 | 1 | 8.3 | 3 | 25.0 | 0 |
|  | AMK | 12 | 8 | 66.7 | 2 | 16.7 | 2 | 16.7 | 0 |
|  | CIP | 12 | 10 | 83.3 | 0 | 0.0 | 2 | 16.7 | 0 |
|  | SXT | 12 | 7 | 58.3 | 0 | 0.0 | 5 | 41.7 | 0 |
|  | NIT | 12 | 8 | 66.7 | 1 | 8.3 | 3 | 25.0 | 0 |
|  | FOS | 0 | 0 |  | 0 |  | 0 |  | 12 |
| Citrobacter freundii complex | AMP | 38 | 22 | 57.9 | 0 | 0.0 | 16 | 42.1 | 0 |
|  | AMC | 38 | 20 | 52.6 | 4 | 10.5 | 14 | 36.8 | 0 |
|  | TZP | 38 | 18 | 47.4 | 4 | 10.5 | 16 | 42.1 | 0 |
|  | CTX | 38 | 20 | 52.6 | 3 | 7.9 | 15 | 39.5 | 0 |
|  | CAZ | 38 | 15 | 39.5 | 3 | 7.9 | 20 | 52.6 | 0 |
|  | FEP | 38 | 17 | 44.7 | 2 | 5.3 | 19 | 50.0 | 0 |
|  | ETP | 38 | 21 | 55.3 | 2 | 5.3 | 15 | 39.5 | 0 |
|  | IPM | 38 | 22 | 57.9 | 2 | 5.3 | 14 | 36.8 | 0 |
|  | MEM | 38 | 19 | 50.0 | 3 | 7.9 | 16 | 42.1 | 0 |
|  | GEN | 38 | 25 | 65.8 | 5 | 13.2 | 8 | 21.1 | 0 |
|  | AMK | 38 | 27 | 71.1 | 4 | 10.5 | 7 | 18.4 | 0 |
|  | CIP | 38 | 18 | 47.4 | 3 | 7.9 | 17 | 44.7 | 0 |
|  | SXT | 38 | 23 | 60.5 | 4 | 10.5 | 11 | 28.9 | 0 |
|  | NIT | 38 | 16 | 42.1 | 6 | 15.8 | 16 | 42.1 | 0 |
|  | FOS | 0 | 0 |  | 0 |  | 0 |  | 38 |
| Serratia marcescens | AMP | 24 | 14 | 58.3 | 4 | 16.7 | 6 | 25.0 | 0 |
|  | AMC | 24 | 10 | 41.7 | 0 | 0.0 | 14 | 58.3 | 0 |
|  | TZP | 24 | 17 | 70.8 | 0 | 0.0 | 7 | 29.2 | 0 |
|  | CTX | 24 | 11 | 45.8 | 1 | 4.2 | 12 | 50.0 | 0 |
|  | CAZ | 24 | 9 | 37.5 | 1 | 4.2 | 14 | 58.3 | 0 |
|  | FEP | 24 | 10 | 41.7 | 2 | 8.3 | 12 | 50.0 | 0 |
|  | ETP | 24 | 16 | 66.7 | 1 | 4.2 | 7 | 29.2 | 0 |
|  | IPM | 24 | 14 | 58.3 | 1 | 4.2 | 9 | 37.5 | 0 |
|  | MEM | 24 | 12 | 50.0 | 3 | 12.5 | 9 | 37.5 | 0 |
|  | GEN | 24 | 16 | 66.7 | 1 | 4.2 | 7 | 29.2 | 0 |
|  | AMK | 24 | 17 | 70.8 | 1 | 4.2 | 6 | 25.0 | 0 |
|  | CIP | 24 | 15 | 62.5 | 3 | 12.5 | 6 | 25.0 | 0 |
|  | SXT | 24 | 16 | 66.7 | 1 | 4.2 | 7 | 29.2 | 0 |
|  | NIT | 0 | 0 |  | 0 |  | 0 |  | 24 |
|  | FOS | 0 | 0 |  | 0 |  | 0 |  | 24 |

S5. Repeat-culture ascertainment, verification-bias description, and persistence-only sensitivity context.

| Characteristic | Repeat culture obtained n=421 | No repeat culture n=542 | p | Metric | Comment |
| --- | --- | --- | --- | --- | --- |
| Episodes, n | 421 | 542 |  | Count | Repeat culture was clinically indicated, not protocol-mandated. |
| Female sex, n (%) | 270 (64.1%) | 345 (63.7%) | 0.878 | Pearson chi-square |  |
| Age, median (IQR) | 55 (36-77) | 56 (38.25-74) | 0.794 | Mann-Whitney U |  |
| Urinary catheter, n (%) | 146 (34.7%) | 172 (31.7%) | 0.335 | Pearson chi-square |  |
| Obstruction, n (%) | 93 (22.1%) | 103 (19.0%) | 0.238 | Pearson chi-square |  |
| Diabetes, n (%) | 137 (32.5%) | 156 (28.8%) | 0.208 | Pearson chi-square |  |
| CKD, n (%) | 73 (17.3%) | 92 (17.0%) | 0.881 | Pearson chi-square |  |
| Sepsis, n (%) | 91 (21.6%) | 126 (23.2%) | 0.548 | Pearson chi-square |  |
| Prior antibiotics within 90 d, n (%) | 181 (43.0%) | 230 (42.4%) | 0.862 | Pearson chi-square |  |
| Polymicrobial infection, n (%) | 85 (20.2%) | 116 (21.4%) | 0.646 | Pearson chi-square |  |
| Co-production positive, n (%) | 42 (10.0%) | 70 (12.9%) | 0.158 | Pearson chi-square |  |
| Biofilm positive, n (%) | 304 (72.2%) | 399 (73.6%) | 0.626 | Pearson chi-square |  |
| Charlson score, median (IQR) | 4 (2-6) | 4 (2-6) | 0.873 | Mann-Whitney U |  |
| Adjudicated treatment failure, n (%) | 140 (33.3%) | 48 (8.9%) | <0.001 | Pearson chi-square | Expected ascertainment difference: repeat culture and microbiological persistence are linked to clinical indication and to the composite outcome; this comparison is descriptive, not causal. |
| Repeat-culture result among cultured episodes | 320 negative; 101 positive | Not cultured |  | Count | Of 101 positive repeat cultures, 80 recovered the same organism and 21 a different organism. |
| Structured reason for no repeat culture | Not applicable | Not recorded |  | Data limitation | Reasons for not obtaining repeat culture were not captured as a structured field. |
| Persistence-only sensitivity | Original coding: 152 failures | Adjudicated: 188 failures |  | Outcome-coding sensitivity | The original-coding model excludes the 36 same-organism persistence-only reclassifications and is reported in S12. |
